# *In Vivo* Transfer and Microevolution of Avian Native IncA/C_2_ *bla*_NDM-1_-Carrying Plasmid pRH-1238 during a Broiler Chicken Infection Study

**DOI:** 10.1128/AAC.02128-17

**Published:** 2018-03-27

**Authors:** Sead Hadziabdic, Jennie Fischer, Burkhard Malorny, Maria Borowiak, Beatriz Guerra, Annemarie Kaesbohrer, Bruno Gonzalez-Zorn, Istvan Szabo

**Affiliations:** a German Federal Institute for Risk Assessment (BfR), Department for Biological Safety, Berlin, Germany; b Departamento de Sanidad Animal and Centro de Vigilancia Sanitaria Veterinaria, Facultad de Veterinaria, Universidad Complutense de Madrid, Madrid, Spain

**Keywords:** antimicrobial resistance, Enterobacteriaceae, Salmonella, NDM-1 carbapenemase, *in vivo* transfer, microevolution, broiler chickens

## Abstract

The emergence and spread of carbapenemase-producing Enterobacteriaceae (CPE) in wildlife and livestock animals pose an important safety concern for public health. With our *in vivo* broiler chicken infection study, we investigated the transfer and experimental microevolution of the *bla*_NDM-1_-carrying IncA/C_2_ plasmid (pRH-1238) introduced by avian native Salmonella enterica subsp. *enterica* serovar Corvallis without inducing antibiotic selection pressure. We evaluated the dependency of the time point of inoculation on donor (*S*. Corvallis [12-SA01738]) and plasmid-free Salmonella recipient [d-tartrate-fermenting (d-Ta^+^) *S*. Paratyphi B (13-SA01617), referred to here as *S*. Paratyphi B (d-Ta^+^)] excretion by quantifying their excretion dynamics. Using plasmid profiling by S1 nuclease-restricted pulsed-field gel electrophoresis, we gained insight into the variability of the native plasmid content among *S*. Corvallis reisolates as well as plasmid acquisition in *S*. Paratyphi B (d-Ta^+^) and the enterobacterial gut microflora. Whole-genome sequencing enabled us to gain an in-depth insight into the microevolution of plasmid pRH-1238 in *S*. Corvallis and enterobacterial recipient isolates. Our study revealed that the fecal excretion of avian native carbapenemase-producing *S*. Corvallis is significantly higher than that of *S*. Paratyphi (d-Ta^+^) and is not hampered by *S*. Paratyphi (d-Ta^+^). Acquisition of pRH-1238 in other Enterobacteriaceae and several events of plasmid pRH-1238 transfer to different Escherichia coli sequence types and Klebsiella pneumoniae demonstrated an interspecies broad host range. Regardless of the microevolutionary structural deletions in pRH-1238, the single carbapenem resistance marker *bla*_NDM-1_ was maintained on pRH-1238 throughout the trial. Furthermore, we showed the importance of the gut E. coli population as a vector of pRH-1238. In a potential scenario of the introduction of NDM-1-producing *S*. Corvallis into a broiler flock, the pRH-1238 plasmid could persist and spread to a broad host range even in the absence of antibiotic pressure.

## INTRODUCTION

Salmonella infections continue to play an important role in veterinary and public health (1). Their importance is nowadays elevated by increased antimicrobial resistance in bacterial populations throughout different stages of food production (2). As carbapenems are members of a potent class of β-lactams and the last option in the treatment of severe human infections, they are not licensed for use in veterinary medicine (3–5). However, reports revealing the appearance of carbapenem-resistant/nonsusceptible bacteria in livestock (poultry, cattle, and swine), food products, wild animals, and the environment have increased in recent years (6–11). The true extent of carbapenemase-producing bacteria in livestock might be underestimated in Europe, due to the voluntary basis for screening at the European Union level (12). One of the most common mechanisms leading to carbapenem resistance is the production of carbapenem-hydrolyzing β-lactamases, mainly encoded by the genes *bla*_VIM_, *bla*_IMP_, and *bla*_NDM_ (which are responsible for the production of class B metallo-β-lactamases), *bla*_KPC_ (class A β-lactamases), and *bla*_OXA-48_ (class D β-lactamases) (13). Worrisome is the worldwide spread of these enzymes by mobile genetic elements, like integrated conjugative elements and plasmids (14). Among carbapenem-resistant/nonsusceptible bacteria, NDM-1-producing bacteria are usually not more virulent. However, due to many nosocomial outbreaks, they are regarded as the most harmful ones. This is linked to the broad geographical reservoirs of NDM-1 in many unrelated bacterial species, due to the location of the *bla*_NDM-1_ genes on broad-host-range plasmids (15). A recent study has revealed the localization of the *bla*_NDM-1_ gene on type 1 IncA/C_2_ plasmid pRH-1238 (referred to as pRH-1738 by Fischer et al. [11]) in an avian native Salmonella enterica subsp. *enterica* serovar Corvallis strain (12-SA01738) isolated from a wild bird (Milvus migrans) in 2012 in Germany (11). The discovery of this first completely sequenced *bla*_NDM-1_-*fosA3*-IncA/C plasmid (GenBank accession number KR091911.1) (16) is of great value due to its host and potential for dissemination into livestock production. This is additionally emphasized by the broad host range of IncA/C plasmids, allowing replication not only in Enterobacteriaceae but also in other bacterial species, such as Pseudomonas and Photobacterium damselae (14). Genome analysis of the pRH-1238 plasmid revealed the coexistence of several resistance genes [*bla*_NDM-1_, *bla*_CMY-16_, *fosA3*, *sul1*, *sul2*, *strA*, *strB*, *aac(6′)-Ib*, *aadA5*, *aphA6*, *tet*(A), *mphA*, *dfrA17*, and *floR*], facilitating resistance to carbapenems, fosfomycins, aminoglycosides, co-trimoxazole, tetracyclines, and macrolides (16). The above-mentioned studies and recent reports on VIM-1-producing Escherichia coli and *S*. Infantis isolates in swine and poultry farms (17, 18) showed that the spread and persistence of carbapenemase-producing bacteria in wild birds and livestock are a reality. With recent reports of VIM-1-producing *S*. Infantis being simultaneously found in swine and minced pork meat in Germany (19), the concerns of human exposure via the food chain are additionally highlighted.

Commercial poultry production is a continuously evolving livestock branch, characterized by fast turnovers and production pressure, which, combined with poor management, could lead to the misusage of antimicrobials (20). This might also favor commercial broiler production acting as a niche for selection of multidrug-resistant bacteria. Furthermore, the intestinal tract of broiler chicken offers a cohabitat for different *Enterobacteriaceae and E. coli*, which is described as a major opportunistic pathogen in chickens with a potential for zoonotic transfer to humans (21). Therefore, it is of relevance to explore if and to what extent different genera and clonal lines might act as potential recipients of the *bla*_NDM-1_-carrying plasmid pRH-1238. This is an important concern due to previous confirmation of the presence of multidrug-resistant and NDM-1-producing *S*. Corvallis bacteria in a wild bird. Knowing the broad host range of *bla*_NDM-1_-carrying plasmids, our aim was to obtain an insight into a potential scenario for this introduction under experimental conditions but conditions that still mimic the rearing management practices common to commercial broiler production. Therefore, we aimed to investigate the intraspecies transfer (Salmonella to Salmonella) and interspecies transfer (Salmonella to endogenous gut microflora) capacities of this *bla*_NDM-1_-carrying plasmid without inducing antibiotic selection pressure in a broiler chicken infection study. The objective of our study was (i) to determine the excretion dynamics of an avian native donor strain (*S*. Corvallis [12-SA01738]) and a poultry-associated recipient strain [d-tartrate-fermenting (d-Ta^+^) *S*. Paratyphi B (13-SA01617), referred to here as *S*. Paratyphi B (d-Ta^+^)] at different inoculation time points, (ii) to analyze the *in vivo* broad-host-range capacity of the pRH-1238 plasmid, and (iii) to analyze the microevolution of the pRH-1238 plasmid (GenBank accession number KR091911.1) using whole-genome sequencing (WGS).

## RESULTS

### Fecal excretion of challenge strains. (i) Group 1.

**FIG 1:**
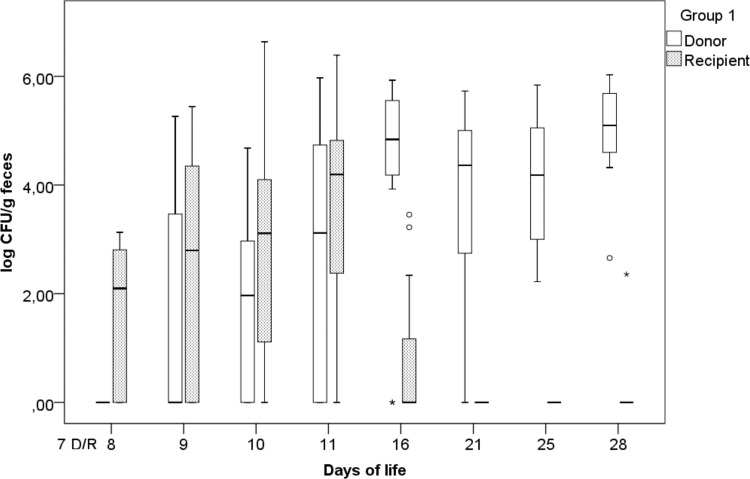
Fecal excretion of the donor (D; *S*. Corvallis) and the recipient [R; *S*. Paratyphi B (d-Ta^+^)] in group 1 (simultaneous inoculation of donor and recipient strains at day 7), expressed as the log number of CFU per gram of feces, with outliers (°) and extreme outliers (*) included.

The fecal excretion of the challenge strains (expressed as the log number of CFU per gram of feces) over time in group 1 (simultaneous inoculation of challenge strains) is represented in Fig. 1. The highest excretion rates for *S*. Corvallis (log 5.09 CFU/g) and *S*. Paratyphi B (d-Ta^+^) (4.19 log CFU/g) were observed on the 28th and 11th days of life, respectively. On the 2nd day postinoculation (p.i.), five animals started to shed *S*. Corvallis, leading up to 11 animals shedding *S*. Corvallis on day 28. As for *S*. Paratyphi B (d-Ta^+^), on the 1st day p.i., seven animals shed *S*. Paratyphi B (d-Ta^+^), with various levels of excretion being detected on the 9th day (seven animals) and 16th day (three animals) of life. Statistical analysis revealed a significant difference between challenge strain excretion on the 8th (*P* = 0.018), 16th (*P* = 0.008), 21st (*P* = 0.008), 25th (*P* = 0.003), and 28th (*P* = 0.003) days of life, contrary to the findings for the 9th (*P* = 0.161), 10th (*P* = 0.285), and 11th (*P* = 0.169) days of life.

### (ii) Group 2.

**FIG 2:**
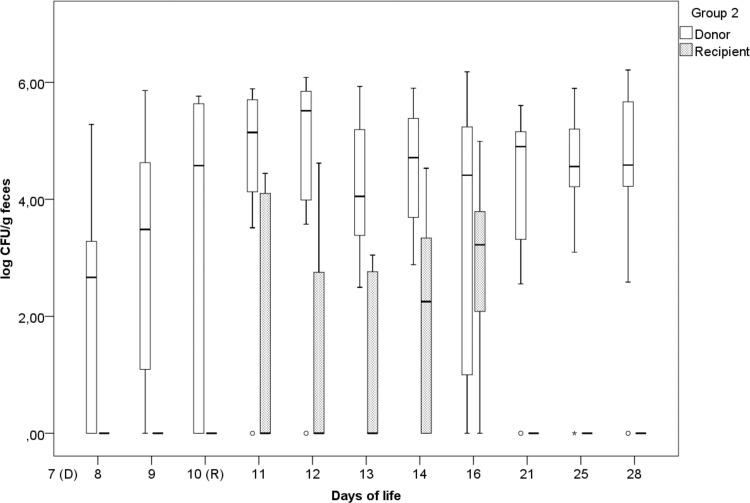
Fecal excretion of the donor (*S*. Corvallis) and the recipient [*S*. Paratyphi B (d-Ta^+^)] in group 2 (delayed recipient inoculation on day 10), expressed as the log number of CFU per gram of feces, with outliers (°) and extreme outliers (*) included.

In group 2 [time-delayed inoculation of *S*. Paratyphi B (d-Ta^+^)], the highest excretion rates for *S*. Corvallis (log 5.51 CFU/g) and *S*. Paratyphi B (d-Ta^+^) (3.22 log CFU/g) were on the 12th and 16th days of life, respectively (Fig. 2). On the 1st day p.i., 6 animals shed *S*. Corvallis, and these animals continued shedding *S*. Corvallis through the 28th day of life, when 10 animals were still excreting *S*. Corvallis. After *S*. Paratyphi B (d-Ta^+^) inoculation on the 10th day of life, four (11th day of life) to nine animals (16th day of life) were excreting *S*. Paratyphi B (d-Ta^+^). In groups 1 and 2, after the 16th day of life, a decrease in *S*. Paratyphi B (d-Ta^+^) excretion was observed (Fig. 1 and 2).

### (iii) Group 3.

**FIG 3:**
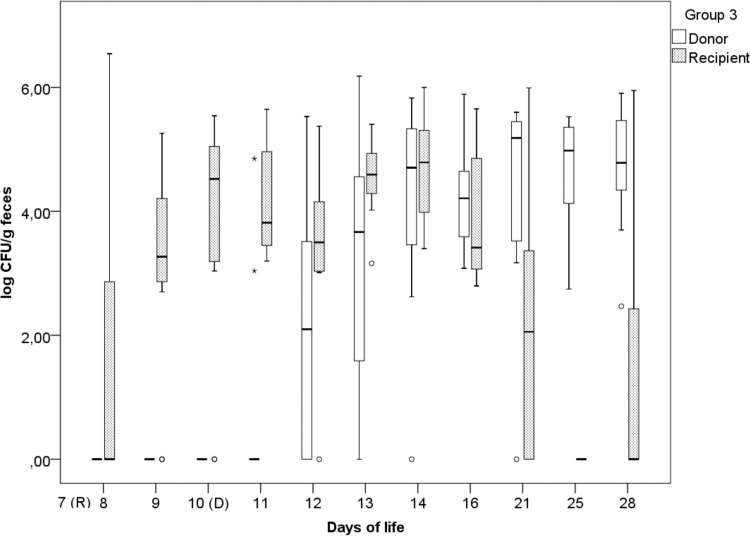
Fecal excretion of the donor (*S*. Corvallis) and the recipient [*S*. Paratyphi B (d-Ta^+^)] in group 3 (delayed donor inoculation on day 10), expressed as the log number of CFU per gram of feces, with outliers (°) and extreme outliers (*) included.

In group 3 (time-delayed inoculation of S. Corvallis), the highest excretion rates for *S*. Paratyphi B (d-Ta^+^) (4.78 log CFU/g) and *S*. Corvallis (log 5.18 CFU/g) were reached on the 14th and 21st days of life, respectively (Fig. 3). In this group, the detection of *S*. Paratyphi B (d-Ta^+^) varied from 3 animals on the 8th day of life to 11 animals on the 16th day of life. Contrary to the results in groups 1 and 2, where a decrease in *S*. Paratyphi B (d-Ta^+^) excretion from the 16th day onwards was observed, seven and four animals were still excreting *S*. Paratyphi B (d-Ta^+^) in this group on the 21st and 28th days of life, respectively. As for *S*. Corvallis, on the 1st day p.i., 2 animals were shedding *S*. Corvallis, and on the 28th day of life 11 animals were shedding *S*. Corvallis.

### Fecal excretion of transconjugants.

In groups 1 and 3, the earliest Enterobacteriaceae transconjugants were detected at 3 days p.i., whereas in group 2, the earliest detection was after 2 days p.i. (Table 1). E. coli transconjugants were detected in all groups, whereas Klebsiella transconjugants were detected only in group 2. During the 21 days p.i., *S*. Paratyphi B (d-Ta^+^) transconjugants were not detected.

**TABLE 1:** Number of animals shedding NDM-1-producing Enterobacteriaceae

Day of life	No. of positive animals		
	Group 1^*a*^	Group 2^*a*^	Group 3^*b*^
8th	0	0	—^*d*^
9th	0	1	—
10th	1	3	—
11th	3	3	0
12th	ND^*c*^	4	0
13th	ND	3	2
14th	ND	1	2
16th	1	3	1
21st	9	4	6
25th	4	4	6
28th	5	2	5

a Inoculation of *S*. Corvallis on the 7th day of life.

b Inoculation of *S*. Corvallis on the 10th day of life.

c ND, not determined.

d —, days prior to *S*. Corvallis inoculation.

### Variability of native plasmid content and plasmid acquisition in challenge strains.

S1 nuclease-restricted pulsed-field gel electrophoresis (S1-PFGE) analysis revealed a higher variability in the native plasmid content of *S*. Corvallis reisolates in groups 1 and 2 than in the native plasmid content of *S*. Corvallis reisolates in group 3 (see Fig. S4 in the supplemental material) as well as the loss of the ∼310-kb IncHI2 and <20-kb ColRNAI plasmids from *S*. Corvallis reisolates in group 1 and 2 (Fig. S2 and S3). In *S*. Corvallis reisolates from all groups, slight deviations (group 1 reisolates G1-28d-T10 and G1-28d-T10 [postmortem], group 2 reisolate G2-16d-T1, and group 3 reisolates G3-28d-T3 and G3-28d-T3 [postmortem], where the reisolates are denoted on the basis of the group [G1 to G3], day of life [1d to 28d], and chick [T1 to T11]) to larger deviations (in group 1 reisolate G1-11d-T10, group 2 reisolates G2-8d-T1 and G2-12d-T1, and group 3 reisolate G3-16d-T3) in the size of the ∼180-kb pRH-1238 plasmid progeny were observed (Fig. S2 to S4). One *S*. Paratyphi B (d-Ta^+^) reisolate (G1-16d-T5) acquired an ∼100-kb plasmid (Fig. S1) lacking *bla*_NDM-1_. Furthermore, hybridization of the *S*. Corvallis reisolates with the NDM-1 probe revealed that the *bla*_NDM-1_ gene was carried by a 110- to 130-kb plasmid (reisolates G1-11d-T10, G2-8d-T1, G2-12d-T1, and G3-16d-T3) and a >400-kb (reisolate G2-28d-T1) plasmid (Fig. S2 to S4) and not the ∼180-kb plasmid.

### Molecular characterization of NDM-1-producing Enterobacteriaceae transconjugants.

In order to assess clonal relatedness as well plasmid(s) acquisition, E. coli and Klebsiella transconjugants were selected for further molecular typing by XbaI restriction analysis and S1-PFGE. In all three experimental groups, macrorestriction with the XbaI endonuclease revealed identical PFGE patterns for NDM-1-producing E. coli transconjugants of phylogenetic group A. At the individual-animal level, different PFGE patterns were observed for NDM-1-producing E. coli transconjugants on a particular sampling day, e.g., the 21st day of life (group 1 reisolates G1-21d-T7-I [E. coli phylogroup A] and G1-21d-T7-II [E. coli phylogroup B1]), as well as different genera of Enterobacteriaceae (resisolates G2-25d-T2-II [E. coli phylogroup D] and G2-25d-T2 -III [Klebsiella pneumoniae]) (Fig. S5). Digestion with S1 nuclease revealed that all selected NDM-1-producing Enterobacteriaceae transconjugants acquired the ∼180-kb pRH-1238 plasmid, with the plasmid contents in E. coli strains that belonged to the same phylogenetic group (e.g., phylogenetic groups D and A) differing (Fig. S6). All strains except one E. coli isolate (G3-21d-environment [subcolony I]) encoded the *bla*_NDM-1_ gene on an ∼180-kb plasmid (Table 2 and Fig. S6).

**TABLE 2:** Molecular characteristics of selected challenge strain reisolates and transconjugants

Species or serovar	Designation^*a*^	Sequence type	Inc designation(s)	% sequence identity^*b*^ to pRH-1238 (size of pRH-1238 progeny [kb^*c*^])	Presence of *bla*_NDM-1_ and *bla*_CMY-16_	Additional resistance gene(s) present
*S*. Corvallis	G1-11d-T10	ST-1541	IncHI2, IncA/C_2_, ColpVC	66.03 (∼100)	Only *bla*_NDM-1_	*bla*_TEM-1B_
*S*. Corvallis	G1-16d-T10	ST-1541	IncHI2, IncA/C_2_, ColRNAI	99.40 (∼180)	Both	
*S*. Corvallis	G1-21d-T10	ST-1541	IncHI2, IncA/C_2_, ColpVC	99.66 (∼180)	Both	*bla*_TEM-1B_
*S*. Corvallis	G1-28d-T10	ST-1541	IncHI2, IncA/C_2_, ColpVC	95.55 (∼170)	Both	*bla*_TEM-1B_
*S*. Corvallis	G1-28d-T10^*d*^	ST-1541	IncHI2, IncA/C_2_, ColRNAI	98.69 (∼180)	Both	
*S*. Corvallis	G2-8d-T1	ST-1541	IncHI2, IncA/C_2_, ColpVC, ColRNAI	79.17 (∼140)	Both	
*S*. Corvallis	G2-10d-T1	ST-1541	IncHI2, IncA/C_2_, ColRNAI	99.54 (∼180)	Both	
*S*. Corvallis	G2-12d-T1	ST-1541	IncHI2, IncA/C_2_, ColpVC, ColRNAI	73.46 (∼130)	Both	
*S*. Corvallis	G2-16d-T1	ST-1541	IncA/C_2_, ColRNAI	95.25 (∼170)	Both	
*S*. Corvallis	G2-25d-T1	ST-1541	IncHI2, IncA/C_2_	99.66 (∼180)	Both	
*S*. Corvallis	G2-28d-T1	ST-1541	IncHI2, IncA/C_2_	99.54 (>400)	Both	
*S*. Corvallis	G3-16d-T3	ST-1541	IncHI2, IncA/C_2_, ColRNAI	75.69 (∼140)	Both	
*S*. Corvallis	G3-28d-T3	ST-1541	IncHI2, IncA/C_2_, ColRNAI	99.45 (∼180)	Both	
*S*. Corvallis	G3-28d-T3^*d*^	ST-1541	IncHI2, IncA/C_2_, ColRNAI	99.65 (∼180)	Both	
E. coli	G1-21d-T7 (I)	ST-2040	IncX1, IncA/C_2_, ColpVC, ColRNAI	99.76 (∼180)	Both	*qnrS1*, *bla*_TEM-1B_
E. coli	G1-21d-T7 (II)	ST-156	IncA/C_2_	99.83 (∼180)	Both	*tet*(B), *bla*_TEM-1B_
E. coli	G1-21d-T1	ST-2040	IncX1, IncA/C_2_, ColpVC, ColRNAI	99.76 (∼180)	Both	*qnrS1*, *bla*_TEM-1B_
E. coli	G2-21d-T5	ST-117	p0111, IncA/C_2_	99.68 (∼180)	Both	
E. coli	G2-21d-T9 (I)	ST-2485	IncA/C_2_	99.85 (∼180)	Both	
E. coli	G2-25d-T2 (II)	ST-2485	IncA/C_2_	98.62 (∼180)	Both	
K. pneumoniae	G2-25d-T2 (III)	ST-1106	Col(MGD2), IncA/C_2_, ColRNAI	99.53 (∼180)	Both	*oqxA*, *oqxB*, *bla*_SHV-1_
E. coli	G3-21d-environment (I)	ST-2040	IncX1, IncA/C_2_, ColpVC, ColRNAI	89.62 (∼170)	Only *bla*_CMY-16_	*qnrS1*, *bla*_TEM-1B_
E. coli	G3-21d-T3	ST-2040	IncX1, IncA/C_2_, ColpVC, ColRNAI	99.74 (∼180)	Both	*qnrS1*, *bla*_TEM-1B_
E. coli	G3-21d-T6 (I)	ST-2040	IncX1, IncA/C_2_, ColpVC, ColRNAI	95.31 (∼180)	Both	*qnrS1*, *bla*_TEM-1B_
*S*. Paratyphi B (d-Ta^+^)	G1-16d-T5	ST-28	Incl1	None	Neither	

a Designations indicate the group (G1 to G3), day of life (1d to 28d), animal (T1 to T11), and subcolony (I to IV) origin.

b Based on consensus sequence length (CLC Genomics Workbench [v9.5] software).

c Based on S1-PFGE restriction.

d Postmortem (cecal content isolates).

### WGS analysis.

With whole-genome sequencing (WGS) analysis, we confirmed the *in vivo* transfer of the pRH-1238 plasmid to different E. coli sequence types (ST) (ST-117, ST-156, ST-2040, and ST-2485) as well as to a K. pneumoniae strain (ST-1106). Furthermore, we reconfirmed the loss of an ∼310-kb IncHI2 plasmid in one *S*. Corvallis reisolate (G2-16d-T1) and the ColRNAI plasmid in several *S*. Corvallis reisolates from group 1 and group 2 (Table 2). The ColRNAI plasmid was detected in E. coli (ST-2040) and K. pneumoniae (ST-1106) strains. On the other hand, the ∼310-kb IncHI2 plasmid was not detected in any of the NDM-1-producing Enterobacteriaceae transconjugants (Table 2). The position of the *bla*_NDM-1_ gene in all *S*. Corvallis reisolates on the pRH-1238 plasmid progeny could be confirmed, whereas one strain (G1-11d-T10) did not harbor the *bla*_CMY-16_ gene (Table 2). One *S*. Corvallis reisolate (G2-28d-T1) harbored a *bla*_NDM-1_-carrying >400-kb plasmid (Fig. S3) (based on Southern blotting and hybridization of S1 nuclease-restricted PFGE). Mapping of this strain to reference plasmid pRH-1238 yielded a consensus sequence identity of 99.53%, and the >400-kb plasmid for this strain might resemble a fusion of IncA/C_2_ (pRH-1238) and IncHI2 (∼490 kb).

Further to the resistome of pRH-1238, additional resistance genes conferring resistance to β-lactams (*bla*_TEM-1B_) were detected in three *S*. Corvallis reisolates (G1-11d-T1, G1-21d-T1, G1-28d-T1), whereas one *S*. Paratyphi B (d-Ta^+^) reisolate (G1-16d-T5) did not harbor additional resistance genes, other than the ∼100-kb Incl1 plasmid. Additional resistance genes for NDM-1-producing Enterobacteriaceae are shown in Table 2.

**FIG 4:**
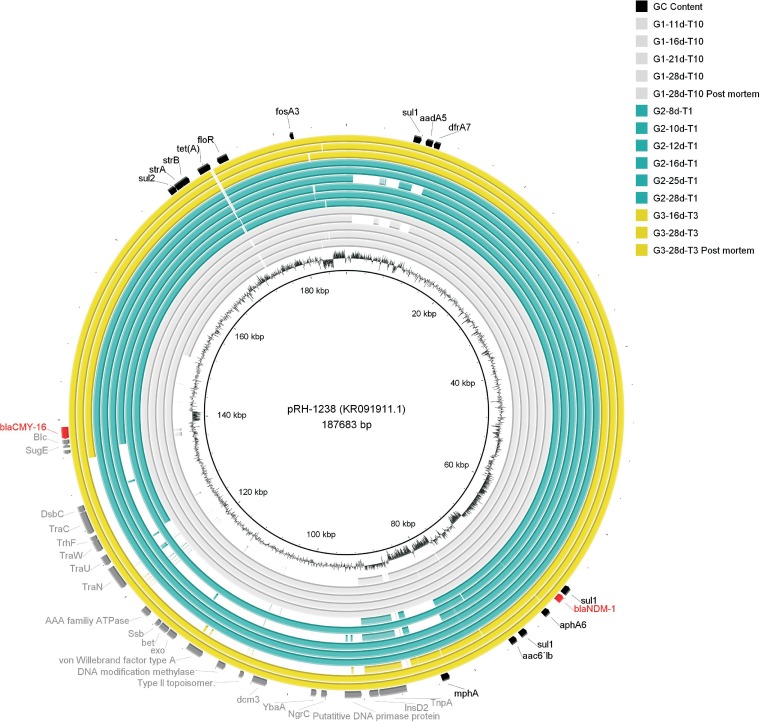
Visualization of assemblies of 14 pRH-1238 consensus sequences from *S*. Corvallis reisolates mapped to the reference pRH-1238 plasmid (GenBank accession number KR091911.1). Innermost circle, pRH-1238 coordinates; second-innermost circle, GC content of pRH-1238 reference plasmid; gray circles, group 1; green circles, group 2; yellow circles, group 3; outermost annotations, the resistome (black), *bla* genes (red), and deletions (dark gray) in Tra1 and the adjacent region, obtained using BRIG (50). Note in strain G1-11d-T10 pRH-1238 progeny the loss of the *bla*_CMY-16_ gene.

**FIG 5:**
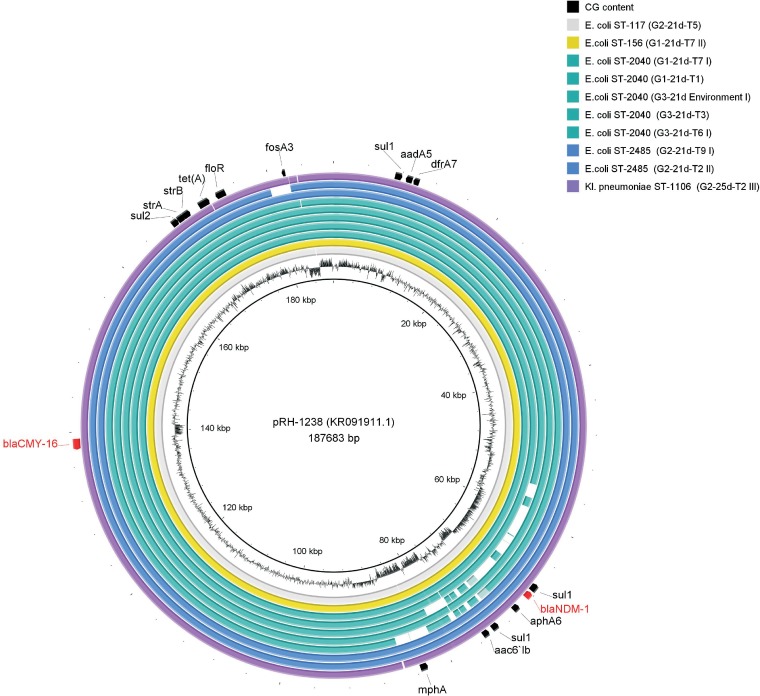
Visualization of assemblies of 10 pRH-1238 consensus sequences from carbapenemase-producing E. coli (ST-117, ST-156, ST-2040, and ST-2485) and K. pneumoniae (ST-1106) transconjugants mapped to the reference pRH-1238 plasmid (GenBank accession number KR091911.1). Innermost circle, pRH-1238 coordinates; second-innermost circle, GC content of pRH-1238 reference plasmid; gray circle, ST-117 strain; yellow circle, ST-156 strain; green circles, ST-2040 strain; blue circles, ST-2485 strain; purple circle, ST-1106 strain; outermost annotations, resistome (black) and *bla* genes (red), obtained using BRIG (50). Note in strain G3-21d-environment (I) pRH-1238 progeny the loss of the *bla*_NDM-1_ gene.

On the basis of consensus sequence mapping of the pRH-1238 plasmid progeny to the reference sequence of pRH-1238 from the *S*. Corvallis reisolates in all groups, a deletion in transfer (Tra) region 1 (Tra1) (∼50 to 60 kb in size) was observed (Fig. 4). In one strain (G1-11d-T10), this led to the loss of *bla*_CMY-16_. Another noteworthy result was the high percent sequence identity among the pRH-1238 progeny from selected Enterobacteriaceae transconjugants, in contrast to *S*. Corvallis reisolates (Table 2 and Fig. 5).

## DISCUSSION

Recent publications have reported the occurrence of carbapenem-nonsusceptible Enterobacteriaceae in wild birds, livestock, and food products and their spread, related to plasmid-mediated carbapenemases (11, 17–19). As carbapenems are not licensed for use in livestock, it is assumed that the occurrence of carbapenemase-producing bacteria is triggered by coselective pressure, since plasmids carrying *bla*_NDM-1_, like the plasmid chosen for use in this study, commonly harbor multiple but variable resistance determinants (16, 22). Still, current research shows that the spread of certain plasmid-mediated resistance genes in broiler chickens is also possible without antibiotic selective pressure (20, 23). Therefore, for understanding the mechanisms contributing to the spread of carbapenem resistance or carbapenem-nonsusceptible isolates *in vivo*, the objective of our animal trial was to explore the broad-host-range capacity and stability of a conjugative *bla*_NDM-1_-carrying plasmid, IncA/C_2_ plasmid pRH-1238, hosted by an *S*. Corvallis strain in chickens without antimicrobial selection pressure, representing the nonuse of carbapenems in livestock. With the help of WGS, such a setup enabled us to obtain an insight into the microevolution of the plasmid *in vivo*.

### Challenge strain excretion.

During our study, we observed prolonged fecal excretion of NDM-1 carbapenemase-producing *S*. Corvallis (12-SA01738), contrary to that of *S*. Paratyphi B (d-Ta^+^) (13-SA01617). Statistical analysis of data from group 1 [simultaneous inoculation of *S*. Corvallis and *S*. Paratyphi B (d-Ta^+^)] revealed that the fecal excretion of *S*. Corvallis was significantly higher toward the end of the trial (the 16th, 21st, 25th, and 28th days of life) (Fig. 1) and was not hampered by the later inoculation of *S*. Paratyphi B (d-Ta^+^), as in group 2 (the difference between *S*. Corvallis excretion in groups 1 and 2 was statistically significant only by 8th day of life) (Fig. 1 and 2). Because of previous studies reporting the invasiveness of *S*. Paratyphi B (d-Ta^+^) toward epithelial cells and macrophages and their presence in ceca, the liver, and the spleen (24), the decreased excretion of *S*. Paratyphi B (d-Ta^+^) observed in our *in vivo* trial is a noteworthy finding. Although this serovar is reported to be broiler associated, we have not observed a competitive advantage, contrary to the findings for *S*. Corvallis. On the other hand, the prolonged excretion of NDM-1-producing *S*. Corvallis in the absence of antibiotic pressure is an important concern due to its resistome and the broad host range of the pRH-1238 plasmid.

### *In vivo* transfer of *bla*_NDM-1_-harboring plasmid pRH-1238.

In our study, we demonstrated the *in vivo* transfer of IncA/C_2_ *bla*_NDM-1_-carrying conjugative plasmid pRH-1238 from avian native *S*. Corvallis to E. coli strains belonging to phylogroups A, B1, and D, represented by four E. coli multilocus sequencing types (ST-117, ST-156, ST-2040, and ST-2485) and a K. pneumoniae isolate (ST-1106) (Table 2). At the individual level, on particular sampling days, we observed pRH-1238 acquisition not only in different E. coli strains but also in different Enterobacteriaceae genera (Table 2). This, together with their rapid onset of excretion (Table 1), demonstrates the broad host range and the high transferability of this multidrug resistance-conferring plasmid, leading to multidrug resistance acquisition in one horizontal gene transfer event. The affected species and genus (E. coli and Klebsiella) underline the importance of this concern due to their clinical relevance and ubiquitous distribution in the environment, acting as potential reservoirs of *bla*_NDM-1_ (25). This deserves attention, especially in commercial broiler production, where contamination pressure due to continuous rearing cycles as well as short interservice breaks could lead to the continuous propagation of pRH-1238 within a mixed bacterial population. With the previous detection of avian native NDM-1 carbapenemase-producing *S*. Corvallis in wild birds, such an entry scenario in commercial broiler production would presumably lead to rapid and diverse *bla*_NDM-1_ dissemination within a broiler flock even without antibiotic pressure. This might also lead to environmental contamination, as has been observed for extended-spectrum β-lactamase (ESBL)/AmpC-producing E. coli strains (26). The broad host range and the high level of transferability without antibiotic pressure should be kept in mind with the implementation of preventative measures. Instead of relying only on selective and coselective pressure as a measure to minimize carbapenemase-producing bacteria, further approaches assessing quantification of resistance gene dissemination with and without selective antibiotic pressure should also be considered.

The detection of enterobacterial transconjugant strains until the end of the trial (in group 1, from the 10th day of life onwards; in group 2, from the 9th day of life onwards; and in group 3, from the 13th day of life onwards) (Table 1) underlines that fact the plasmid acquisition has a presumably low or negligible fitness cost (27). Intestinal bacteria serve as reservoirs or even vectors for antibiotic resistance plasmids (28), findings which are further emphasized by the plasmid and resistance gene acquisition from the gut microflora observed in challenge strains (Table 2).

As we did not detect NDM-1-producing *S*. Paratyphi B (d-Ta^+^) transconjugants, we assume that the host's E. coli population has an important influence on the reception and further spread of the pRH-1238 plasmid. This might be linked to the dense and diverse population and host gut adaptation of E. coli, serving as native recipients of pRH-1238. Although our *in vitro* filter mating conjugation experiments indicated a high transfer rate of the pRH-1238 plasmid to *S*. Paratyphi B (d-Ta^+^) (see Table S2 in the supplemental material), its absence *in vivo* might be linked to (i) serovar colonization dynamics, (ii) the abundance, diversity, and interference of E. coli strains, and (iii) the detection limit of the method used in this study (∼100 CFU/g). The majority of E. coli NDM-1 producers belonged to phylogroup A (represented by ST-2040); however, ST-117 and ST-156 strains were also detected. Besides being associated with poultry, strains of these STs are also described to be a potential source of not only β-lactam resistance genes but also polymyxin resistance genes (29–31). In a recent publication, a human-acquired *mcr-1*-carrying ST-117 strain of avian origin was characterized, highlighting the capability of this ST for resistance gene acquisition (30). The observed dominance of E. coli strains belonging to phylogroup A might resemble their occurrence in the gut or their ability to serve as native recipients for pRH-1238, as described for certain clonal lines dominant in the spread of the plasmid-mediated *oqxAB* gene encoding quinolone resistance (23).

Furthermore, *bla*_CMY-16_ is a variant of the *bla*_CMY-2_ lineage, which has been described to be the most common plasmid-mediated AmpC enzyme common to different Enterobacteriaceae worldwide (32). Therefore, the introduction of the pRH-1238 plasmid into a broiler flock should be assessed as well in light of the potential further dissemination of not only *bla*_NDM-1_ but also *bla*_CMY-16_, which might be additionally propagated due to the use of cephalosporins in commercial poultry production. For future understanding, it is of interest to predict the dissemination potential of plasmid-mediated resistance genes relevant to public health and questioning the genera or serovars dominant in this exchange. Such data could contribute to a wider picture, broaden our knowledge for carbapenem resistance risk assessment, and serve as an asset for future approaches minimizing the spread of antimicrobial resistance *in vivo*.

### Plasmid content variability in S. Corvallis reisolates.

The observed native plasmid variability (plasmids of ∼310 kb [IncHI2], 180 kb pRH-1238 [IncA/C_2_], and <20 kb [ColRNAI]) was predominant in *S*. Corvallis reisolates from groups 1 and 2 (Fig. S2 and S3). This observation leads us to the assumption that the simultaneous and initial inoculation of *S*. Corvallis led to certain rearrangement mechanisms in native plasmid content, observed as the complete loss of the ∼310-kb IncHI2 and <20-kb ColRNAI plasmids or partial region deletions in the ∼310-kb IncHI2 plasmid (up to ∼100 kb) and in the ∼180-kb pRH-1238 plasmid (up to ∼50 to 60 kb) (Fig. S2 to S4). We speculate that the earlier (7th day of life) inoculation of *S*. Corvallis in experimental groups 1 and 2 led to this occurrence. In a recent study by Card et al. (33) with a chemostat which mimicked the broiler microbiome, it seemed that the bacterial community stabilized by day 6. In our case, this unstable microbial population might support mobilome restructuring as well the interaction and subsequent acquisition of *bla*_TEM-1B_ in *S*. Corvallis reisolates from group 1 in later stages of the trial (Table 2). Plasmid exchange and certain structural deletions might also be an important part of host adaptation regulation. Previous studies have reported that the acquisition and loss of certain genetic elements in bacteria are stimulated by the adaptation to the new environment, which influences their pathogenicity and might have subsequent consequences for human and animal health (34, 35). As our study focused on NDM-producing Enterobacteriaceae detectable on xylose-lysine-deoxycholate (XLD) and chromID Carba agar and we did not conduct metagenomics analysis, we presume that *bla*_TEM-1B_ might have originated from an E. coli ST-2040 strain. Furthermore, it seems that this sequence type played a significant role in plasmid exchanges (acquisition of pRH-1238 and ColRNAI and transfer of the ColpVC plasmid) with *S*. Corvallis (Table 2).

### Microevolution of pRH-1238 in *S*. Corvallis and enterobacterial transconjugants.

Besides the plasmid content variability observed after S1 restriction for the IncHI2 (∼310-kb) and ColRNAI (<20-kb) plasmids in *S*. Corvallis reisolates, the large-scale structural changes in pRH-1238 progeny were determined as deletions in Tra1 and downstream (∼50 to 60 kb in size) between two resistance islands: ARI-A [harboring *sul1*, *bla*_NDM-1_, *aph6*, *mphA*, and *aac6′lb*] and ARI-B [harboring *sul2*, *strA*, *strB*, *tet*(*A*), *floR*, *fosA3*, *sul1*, *aadA5*, *dfrA7*] (16). These deletions did not lead to a significant alteration of the pRH-1238 β-lactam resistome, as only one strain did not harbor *bla*_CMY-16_ (Table 2 and Fig. 4), due to its position adjacent to Tra1 of pRH-1238. This occurrence is in line with observations indicating the large-scale structural changes often observed in neighboring areas of transposons and insertion sequence elements, indicating that these elements contribute to plasmid genome evolution (36). In a recent *in vitro* study by Porse et al. (37), deletions in the IncN plasmid (also constituting Tra regions) in recipient E. coli strains were observed, contrary to the findings for native K. pneumoniae and recipient Klebsiella strains. The authors stated that this occurrence might possess a potential competitive benefit for recipient E. coli strains. In contrast to the findings of our *in vivo* study, the deletions in the pRH-1238 progeny were dominant in *S*. Corvallis reisolates and not E. coli and K. pneumoniae strains (Fig. 4 and 5), suggesting that these deletions might be host or incompatibility group dependent. Generally, the observed losses of the IncHI2 and ColRNAI plasmids as well as deletions in the pRH-1238 progeny might indicate an evolutionary background in *S*. Corvallis adaptation which enables maintenance of the pRH-1238 resistome even without antibiotic pressure in wild birds.

A noteworthy observation was a >400-kb plasmid in sample G2-28d-T1 (Table 2 and Fig. S3) which seemed to be a fusion of IncHI2 and IncA/C_2_ plasmid pRH-1238. This mobilome restructuring might be triggered by intrinsic *S*. Corvallis mechanisms and also linked with the persistence of *bla*_NDM-1_ in *S*. Corvallis. Namely, plasmid fusion and cointegration are frequent phenomena in plasmid evolution and adaptation and prevent, e.g., plasmid incompatibility and facilitate the interaction with a broad range of hosts (38). For a better understanding, it is of interest to explore if these occurrences are triggered by certain metabolic processes in the gut, bacterial stress, or a possible interaction with competitive gut microflora. Interestingly, pRH-1238 progeny from two strains sampled from the cecal contents showed a high percentage of sequence identity to the pRH-1238 backbone (Table 2 and Fig. 4). Such an occurrence indicates that the *S*. Corvallis reisolates harboring native pRH-1238 exist in the intestinal tract and continuously disseminate pRH-1238 *in vivo*. Previous findings have reported on the higher level of colonization of Salmonella in the ceca, leading to higher rates of conjugation, which has been observed for a conjugative extended-spectrum cephalosporin resistance gene-harboring plasmid from *S*. Newport to E. coli strains and vice versa (39). Furthermore, deletions in pRH-1238 among Enterobacteriaceae transconjugants were minor and not attributed to Tra1, and the sequences of pRH-1238 progeny with these deletions revealed a higher degree of identity to the reference backbone of pRH-1238 (Table 2 and Fig. 5). This indicates that the pRH-1238 acquisition or transfer process itself might not lead to a significant alteration of pRH-1238 in transconjugant strains and that these strains might also serve as long-term reservoirs of pRH-1238 *in vivo*.

In conclusion, we demonstrated the prolonged fecal excretion of an avian native NDM-1 carbapenemase-producing *S*. Corvallis strain (12-SA01738) with microevolutionary deletions in the pRH-1238 backbone that preserved the *bla*_NDM-1_ gene during a broiler chicken *in vivo* study. The conjugative pRH-1238 IncA/C_2_ *bla*_NDM-1_-carrying plasmid was transferable to different Enterobacteriaceae, expanding its resistance gene pool among gut microflora in the absence of antibiotic pressure throughout the trial. This study shows at the molecular level how the rapid and diverse dissemination of *bla*_NDM-1_-harboring IncA/C_2_ plasmids in commercial broiler production can occur even in the absence of selective pressure. Furthermore, it highlights the need for understanding the mechanisms of the interaction of the host microflora and Salmonella serovars and calls for additional efforts in future intervention approaches to avoid the further spread of multidrug resistance plasmids in commercial broiler production.

## MATERIALS AND METHODS

### Challenge strains.

Avian native Salmonella enterica subsp. *enterica* serovar Corvallis (strain 12-SA01738) of ST-1541 harboring the *bla*_NDM-1_-carrying IncA/C_2_ plasmid pRH-1238 (GenBank accession number KR091911.1) was selected as the donor strain. Native d-tartrate-fermenting (d-Ta^+^) Salmonella enterica subsp. enterica serovar Paratyphi B (13-SA01617), referred to here as *S*. Paratyphi B (d-Ta^+^), of ST-28, isolated in 2013, with intrinsic resistance to nalidixic acid was selected as the recipient. The pRH-1238 plasmid is the first completely sequenced *bla*_NDM-1_-*fosA3*-IncA/C plasmid. It is 187,683 bp in size, has a GC content of 51.7%, and contains 173 predicted coding sequences (CDSs). It contains two resistance islands (ARI-A and ARI-B) and two transfer (Tra) regions (Tra1 and Tra2), with *bla*_NDM-1_ being located in ARI-A and *bla*_CMY-16_ being located in Tra1 (16). Besides pRH-1238, the donor strain harbors two additional plasmids of incompatibility group IncHI2 (∼310 kb) and ColRNAI (<20 kb), whereas *S*. Paratyphi B (d-Ta^+^) was selected as plasmid-free recipient strain. The phenotypic and genotypic properties of the donor and recipient strains are listed in Table S1 in the supplemental material. The selection of *S*. Paratyphi B (d-Ta^+^) was based on its high prevalence in commercial poultry production in Germany (40) as well as optimal *in vitro* conjugation transfer frequency (CTF) at 42°C (which corresponds to the average body temperature of birds) with *S*. Corvallis as the donor strain (Table S2). All strains were obtained from the strain collection of the National Reference Laboratory (NRL) for Salmonella in Germany.

### *In vitro* filter mating conjugation experiments.

Prior to our *in vivo* study, *in vitro* filter mating conjugation experiments with selected Salmonella strains (Table S2) were conducted to determine the average conjugation transfer frequency (CTF) for four potential recipient strains with *S*. Corvallis (12-SA01738) as the donor. After aerobic growth with gentle shaking at 37°C to obtain an optical density at 560 nm (OD_560_) value of 0.25, a mixture of the Salmonella donor and recipient at a ratio of 1 to 2 was centrifuged (20,000 × *g* for 2 min), inoculated on 0.45-μm -pore-size filter membranes (Merck Millipore, Germany) that had previously been placed on lysogeny agar (LBA; Thermo Fisher Scientific, Germany), and incubated for 4 h at room temperature (RT), 37°C, or 42°C. Following incubation, the filter membranes were suspended in 4 ml of lysogeny broth (LBL; Thermo Fisher Scientific, Germany), decimally diluted, and plated on transconjugant selective plates (as described in Table S3). All filter mating conjugation experiments were conducted in triplicate in order to determine the average CTF rate (Table S2).

### Broiler chicken infection study.

**FIG 6:**
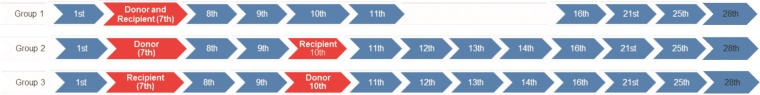
Experimental design of the test groups (groups 1 to 3), with inoculation (red) and sampling (blue) days, as well as the end day of the experiment (blue with black numbers), being marked.

For the *in vivo* trial, 33 broiler chicks (Ross 308) were randomly selected as 1-day-old chicks, without prior determination of the chick sex. Housing, clinical examination, individual labeling, and sampling followed. Animals were randomly divided into three experimental groups (group 1 [G1], G2, and G3), each containing 11 animals (animals T1 to T11) and housed in the facilities for animal experiments at the German Federal Institute for Risk Assessment, Berlin, Germany. In order to evaluate the dependency of the time point of inoculation on excretion of the challenge strains for the 28 days of the experiment, three experimental setups (groups 1, 2, and 3) were assembled. In group 1, the donor and recipient were simultaneously inoculated on the 7th day of life, whereas in group 2 (inoculation on the 7th day of life for the donor and the 10th day of life for the recipient) and group 3 (inoculation on the 7th day of life for the recipient and the 10th day of life for the donor), time-delayed inoculations were used. At the end of the experiment (at the 28th day of life), all animals were handled carefully following electrical stunning before being sacrificed for postmortem cecum extirpation. The experimental design containing the time frame and the related activities is shown in Fig. 6. During the experiment, microambient conditions complied with the hybrid management guide, and the animals were checked daily for evaluation of criteria for health and well-being. The animal trials were approved by the German State Authority for Health and Social Affairs (Lageso; no. 0308/15).

To prevent unintentional cross-reaction with intestinal microbiota, 1-day-old chicks were tested for possible colonization with (i) ESBL/pAmpC- or carbapenemase-producing E. coli using the laboratory protocol provided and recommended by the EURL for antimicrobial resistance (41) and (ii) Salmonella spp. following standard ISO 6579:2002/Amd 1:2007 (International Organization for Standardization, Switzerland). The procedure was repeated on the day of inoculation to reconfirm the absence of interfering background flora.

### Inoculation challenge and sampling plan.

On the day of inoculation, both challenge strains were grown aerobically in LBL at 37°C with gentle shaking to obtain an OD_560_ value of 0.35, which corresponded to a bacterial count of 4 × 10^6^ CFU per 100 μl for both strains used as the inoculum. On day 7, animals were orally inoculated (for group 1, with the donor and recipient strains; for group 2 with the donor strain; for group 3, with the recipient strain), followed by a second inoculation (for group 2, with the recipient strain; for group 3, with the donor strain) on the 10th day of life. After inoculation, a 4-day consecutive sampling was performed, and further sampling was performed two times per week toward the end of trial (Fig. 6). Animals were always sampled individually in a particular time frame with preweighed cotton cloacal swabs (Deltalab, Spain) in order to determine the counts of the excreted challenge strains, expressed as the number of CFU per gram of feces.

### Bacterial strain isolation.

After suspending the fecal material (∼0.2 g) in 5 ml of 0.85% (wt/vol) NaCl, the suspension was subjected to decimal dilution and a 100-μl deposition volume per plate was plated with an automatic spiral plater in duplicate on selective agar plates using the spiral colony counting technique with a Whitley automatic spiral plater (Don Whitley Scientific, UK). On the 1st day postinoculation (p.i.), dilutions of 1:10 and 1:10^3^ were plated, and these were later adjusted to 1:10 and 1:10^2^ on the basis of excretion dynamics. Challenge strain and transconjugant detection was based on growth on xylose-lysine-deoxycholate (XLD) agar (Thermo Fisher Scientific, Germany) with antibiotic supplementation (meropenem [0.125 mg/liter], cefotaxime [1 mg/liter], and/or nalidixic acid [50 mg/liter]), depending on the target strain (donor, recipient, or Salmonella transconjugants), and chromID Carba (bioMérieux, France) for detection of carbapenemase-producing Enterobacteriaceae (CPE) (Table S3). Colonies suspected of being Salmonella were detected on XLD agar as red-yellow colonies with a black center, and CPE (e.g., E. coli, Klebsiellae) were detected on chromID Carba as purple and blue colonies. In order to further characterize the challenge strain [e.g., to characterize the variability in the plasmid content in *S*. Corvallis and plasmid acquisition in *S*. Paratyphi B (d-Ta^+^)], reisolates from particular chicks within each group [*S*. Corvallis reisolates in group 1 (chick T10; see below), group 2 (chick T1), and group 3 (chick T3) and *S*. Paratyphi B (d-Ta^+^) reisolates in group 1 (chick T5), group 2 (chick T1), and group 3 (chick T3)] were preserved, whereas when possible four subcolonies (marked I to IV) of presumptive NDM-1 carbapenemase-producing Enterobacteriaceae transconjugants were selected for molecular characterization. Selected strains were denoted on the basis of the group (G1 to G3), day of life (1d to 28d), chick (T1 to T11), and subcolony (I to IV) origin.

### Confirmation of presence of pRH-1238 in transconjugants.

Transconjugants were screened by PCR amplification of *bla*_NDM-1_ and *bla*_CMY-16_ using a 1:10-diluted overnight culture as the template as described in previous publications (42, 43). PCR mixtures (25-μl reaction volume) contained 17.5 μl of master mix (4 μl primer mix [each at 400 nM], 2.5 μl deoxynucleoside triphosphate [dNTP] mix [each 200 μM], 2.5 μl 10× buffer, 1.25 μl MgCl_2_ [2.5 mM], 7.05 μl PCR-grade water, 0.2 μl *Taq* DNA polymerase [Invitrogen, USA]) and 7.5 μl DNA template under the following conditions: initialization for 5 min at 94°C; denaturation, annealing, and extension for 30 s at 94°C, 30 s at 54°C, and 1 min at 72°C for 30 cycles; elongation for 5 min at 72°C; and a final hold at 4°C.

### Genus/species identification of transconjugants through MALDI-TOF MS.

A random selection of phenotypically different (purple and blue) colonies of Enterobacteriaceae transconjugants grown on chromID Carba was tested by matrix-assisted laser desorption ionization–time of flight mass spectrometry (MALDI-TOF MS; Bruker Daltonik, Germany) to confirm the taxonomic classification at the genus and species levels. After subculture on LBA, a small amount of a bacterial colony was transferred in duplicate onto the target wells of an MSP 96 polished steel barcode (BC) plate (Bruker Daltonik, Germany) and suspended in 1 μl of HCCA (α-cyano-4-hydroxycinnamic acid) matrix (Bruker Daltonik, Germany) according to the manufacturer's instructions. After the bacteria were air dried at room temperature, mass spectrometry was performed using MALDI flexControl software (Bruker Daltonik, Germany), and microorganisms were identified according to the values obtained, where values of 2.3 to 3.0, obtained by use of the MALDI Biotyper (Bruker Daltonik, Germany) database, indicated a very sure species identification, values of 2.0 to 2.29 indicated a sure genus identification and a probable species identification, values of 1.7 to 1.99 indicated a probable genus identification, and values of 0.0 to 1.69 indicated a not reliable identification.

### Molecular characterization of transconjugants. (i) Phylotyping of E. coli transconjugants.

A multiplex PCR assay for phylotyping of 104 selected E. coli transconjugants (for group 1, *n* = 43; for group 2, *n* = 19; for group 3, *n* = 42) into phylogenetic groups A, B1, B2, and D was conducted (44). All PCRs were done in a 25-μl reaction volume containing 18 μl of master mix (5 μl primer mix [10 pmol each primer], 2.5 μl dNTP mix [each 200 μM], 2.5 μl 10× buffer, 7.5 μl PCR-grade water, 0.5 μl *Taq* DNA polymerase [Invitrogen, USA]) and 7 μl of the DNA template (a 1:10-diluted overnight culture) under the following conditions: an initialization step of 5 min at 94°C; denaturation, annealing, and extension for 30 s at 94°C, 30 s at 65°C, and 30 s at 72°C for 30 cycles; elongation for 5 min at 72°C; and a final hold at 4°C.

### (ii) XbaI PFGE analysis.

Macrorestriction with the XbaI endonuclease (Roche Applied Sciences, Switzerland) was performed for 104 NDM-1-producing Enterobacteriaceae transconjugant strains according to the PulseNet standardized protocol (www.pulsenetinternational.org) (45) using a contour-clamped homogeneous electric field (CHEF-DRIII) system (Bio-Rad Laboratories, Madrid, Spain) for separation of the fragments. As a molecular size standard, Salmonella serovar Braenderup strain H9812 (restricted with XbaI) was used. Gel imaging was conducted in a GenBox apparatus (Syngene, UK), and gel documentation was conducted with GeneSnap software (Syngene, UK).

### Molecular characterization of the pRH-1238 plasmid. (i) S1-PFGE plasmid profiling.

In order to evaluate the variability in native plasmid content (the IncHI2 plasmid [∼310 kb], IncA/C_2_ plasmid pRH-1238 [187,683 bp], and the ColRNAI plasmid [<20 kb]) among the *S*. Corvallis strains and plasmid acquisition in *S*. Paratyphi B (d-Ta^+^), in total 20 reisolates (for group 1, animal T10 [*n* = 6]; for group 2, animal T1 [*n* = 8]; for group 3, animal T3 [*n* = 6]) and 18 reisolates (for group 1, animal T5 [*n* = 4]; for group 2, animal T1 [*n* = 6]; for group 3, animal T3 [*n* = 8]), respectively, were typed by S1-PFGE. Additionally, 104 NDM-1-producing Enterobacteriaceae transconjugants were typed by S1 nuclease (TaKaRa, USA) restriction in order to visualize the transferred ∼180-kb pRH-1238 plasmid. For S1-PFGE gels intended for Southern blotting, a MidRange pulsed-field gel marker (Biolabs, USA) was used as a size marker. The generated fragments were separated using the CHEF-DRIII system (Bio-Rad Laboratories, Spain) with S-1 running conditions (1 s to 25 s, 17 h, 6 V/cm, 120 V), as previously described (46).

### (ii) Southern blotting of an S1-PFGE gel hybridized with a *bla*_NDM-1_ probe.

To map the position of a single carbapenem resistance marker (the *bla*_NDM-1_ gene on pRH-1238), Southern blotting and hybridization of an S1-PFGE gel with a digoxigenin-labeled NDM-1 probe of 20 selected *S*. Corvallis reisolates and 16 NDM-1-producing Enterobacteriaceae isolates (group 1, *n* = 5; group 2, *n* = 6; group 3, *n* = 5) belonging to different E. coli phylogroups as well as one K. pneumoniae were conducted as previously described (46).

### (iii) Whole-genome sequencing analysis.

On the basis of the variability of the plasmid content detected with S1-PFGE analysis, the genomes of 25 strains, including 15 challenge strain reisolates [*S*. Corvallis, *n* = 14; *S*. Paratyphi B (d-Ta^+^), *n* = 1] and 10 NDM-1-producing Enterobacteriaceae transconjugants, were sequenced. All strains selected for whole-genome sequencing are listed in Table 2.

Genomic DNA was extracted using a PureLink genomic DNA minikit (Invitrogen, USA), followed by fluorometric DNA concentration (in nanograms per microliter) measurement by Qubit fluorometric quantitation (Invitrogen). Sequencing libraries were prepared using a Nextera XT DNA sample preparation kit (Illumina, San Diego, CA, USA) according to the manufacturer's protocol. Paired-end sequencing was performed on an Illumina MiSeq benchtop apparatus (MiSeq reagent [v3] 600 cycle kit; 2 × 300 cycles). Raw reads were assembled *de novo* using CLC Genomics Workbench (v9.5) software (Qiagen, Denmark), and STs, plasmid content, and resistance genes were detected using the services BatchUploader (47), PlasmidFinder (48), and ResFinder (49), available at the Center for Genomic Epidemiology (CGE; http://www.genomicepidemiology.org).

In order to evaluate microevolutionary changes in the *bla*_NDM-1_-carrying pRH-1238 plasmid among *S*. Corvallis reisolates and to assess possible structural deletions in the plasmid backbone due to the transfer in Enterobacteriaceae transconjugants, assembled genomes were mapped against the reference sequence of pRH-1238 (size, 187,683 bp; GenBank accession number KR091911.1) using CLC Genomics Workbench (v9.5) software. The percent sequence identity was calculated on the basis of the size of the consensus sequence in reference to that of the sequence of pRH-1238, whereas their visualization was done using BLAST Ring Image Generator (v0.95; BRIG) software (50).

### Statistical analysis of challenge strain excretion.

For statistical analysis of challenge strain fecal excretion, SPSS21 (v2.0) software (SPSS Inc., USA) was used. Due to the nonnormal distribution of bacterial count excretion data, a log transformation was conducted. In experimental group 1 (simultaneous inoculation of donor and recipient strains), excretion of challenge strains was compared using the Wilcoxon matched-pairs test on the basis of the related data assumption, and comparisons between groups were conducted by the Mann-Whitney U test. Differences were considered significant if the *P* value was less than or equal to 0.05. Challenge strain excretion is graphically presented by box-whisker plots. The boxes indicate the medians (horizontal lines) and the lower and upper quartiles (lower and upper sides of the boxes, respectively). Outliers, which are values numerically distant from the rest of the data, were included for determination of the statistical significance.

## Supplementary Material

Supplemental material
