# Supplementary material for: In Vivo Transfer and Microevolution of Avian Native IncA/C2 blaNDM-1-Carrying Plasmid pRH-1238 during a Broiler Chicken Infection Study
Source: Antimicrob Agents Chemother. 2018 Mar 27;62(4):e02128-17. doi: 10.1128/AAC.02128-17 (PMC5913973; doi:10.1128/AAC.02128-17)
Supplement: Supplemental material [file supp_62_4_e02128-17__index.html]

In Vivo Transfer and Microevolution of Avian Native IncA/C2 blaNDM-1-Carrying Plasmid pRH-1238 during a Broiler Chicken Infection Study — Supplemental material 

# *In Vivo* Transfer and Microevolution of Avian Native IncA/C2 *bla*NDM-1-Carrying Plasmid pRH-1238 during a Broiler Chicken Infection Study

## Supplemental material

- Supplemental file 1 -

  Supplemental Tables S1 to S3 and Figures S1 to S6

  PDF, 508K
