# Supplementary material for: In Vivo Transfer and Microevolution of Avian Native IncA/C2 blaNDM-1-Carrying Plasmid pRH-1238 during a Broiler Chicken Infection Study
Source: Antimicrob Agents Chemother. 2018 Mar 27;62(4):e02128-17. doi: 10.1128/AAC.02128-17 (PMC5913973; doi:10.1128/AAC.02128-17)
Supplement: Supplemental material [file AAC.02128-17_zac004187054s1.pdf]

TABLE S1. Phenotypic and genotypic properties of challenge strains

	Challenge strains	Origin/ Year of isolation	Plasmids	Phenotypic resistance profile (by MIC)a)	Genotypic resistance profile of pRH-1238	Genotypic resistance of others	Reference
**Donor**	NDM-1 producing *S.* Corvallis (12-SA01738)	Black kite (*Milvus migrans*) 2012	IncHI2 (~310 kb)<br>IncA/C$_2$ pRH-1238 (187,683 bp)<br>ColRNAI (< 20 kb)	AMP, CHL, CIP, FFN, FOT, KAN SMX, STR, TAZ, TET, TMP	*bla*$_{CMY-16}$, *bla*$_{NDM-1}$, *floR, accA4* [*aac(6´)-Ib*], *tet(A), drfA1/7/17, strA/B, sul1, sul2, aadA5, fosA3, aphA6, mphA*	*qnrS*	(1, 2)
**Recipient**	*S.* Paratyphi B (*d*Ta+) (13-SA01617)	Chicken meat 2013	none	CIP, NAL, STR, TMP	-	*aadA1, dfrA1*	-

[a] MIC carried out following CLSI guidelines, CLSI M07-A9 and using EUCAST epidemiological cut-off values (http://www.eucast.org)

Definition of abbreviations:

AMP (Ampicillin), CHL (Chloramphenicol), CIP (Ciprofloxacin), NAL (Nalidixic acid), FFN (Florfenicol), FOT (Cephotaxime),

KAN (Kanamycin), SMX (Sulphamethoxazole), STR (Streptomycin), TAZ (Cephtazidime), TET (Tetracycline), TMP (Trimethoprim)

TABLE S2. Average CTF rates based on filter mating conjugation experiments for donor and recipient strains

**Donor**	**Recipients**	**Strain no.**	**Conjugation transfer frequency (CTF) rate**		
			RT	37°C	42°C
NDM-1 producing *S*. Corvallis (12-SA01738)	*S*. Enteritidis [with 60 kb *pSEV* virulence plasmid (3)]	07-SA03428	$5,08 \times 10^{-5}$	$8,71 \times 10^{-3}$	$1,88 \times 10^{-2}$
	S. Enteritidis (without 60 kb *pSEV* virulence plasmid)	04-SA02117	$8,56 \times 10^{-5}$	$1,50 \times 10^{-2}$	$1,57 \times 10^{-2}$
	S. Infantis	14-SA03263	$6,64 \times 10^{-5}$	$9,04 \times 10^{-3}$	$1,10 \times 10^{-2}$
	S. Paratyphi B (*d*Ta+)	13-SA01617	$3,50 \times 10^{-4}$	$8,07 \times 10^{-3}$	$1,06 \times 10^{-2}$

TABLE S3. Antibiotic supplementation of selective plates for target microorganisms

Target microorganisms	Medium	Antibiotic supplementation		
		Cefotaxime (1 mg/l)	Meropenem (0.125 mg/l)	Nalidixic acid (50 mg/l)
S. Corvallis	XLD agar	+	+	-
S. Paratyphi B (*d*Ta+)	XLD agar	-	-	+
NDM-1-producing *S.* Paratyphi B (*d*Ta+)	XLD agar	+	+	+
Carbapenemase-producing Enterobacteriaceae (CPE)	chromID® CARBA agar	unknown	unknown	unknown

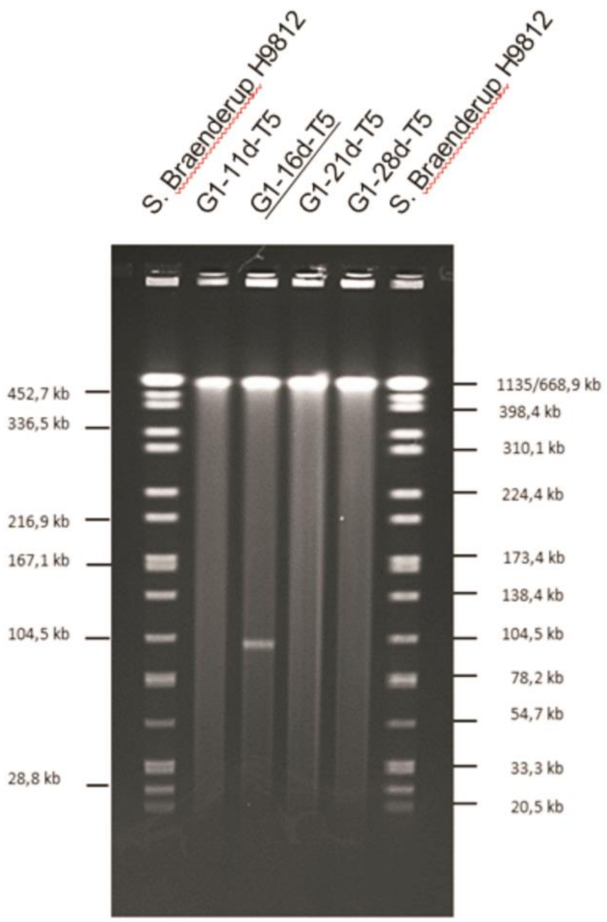

FIGURE S1. S1-PFGE analysis of *S*. Paratyphi B (*d*Ta+) reisolates from Group 1 in lanes 2-5 with molecular-sized standard, *Salmonella* Braenderup strain H9812 (restricted with XbaI) in lanes 1 and 6. Underlined strain selected for WGS.

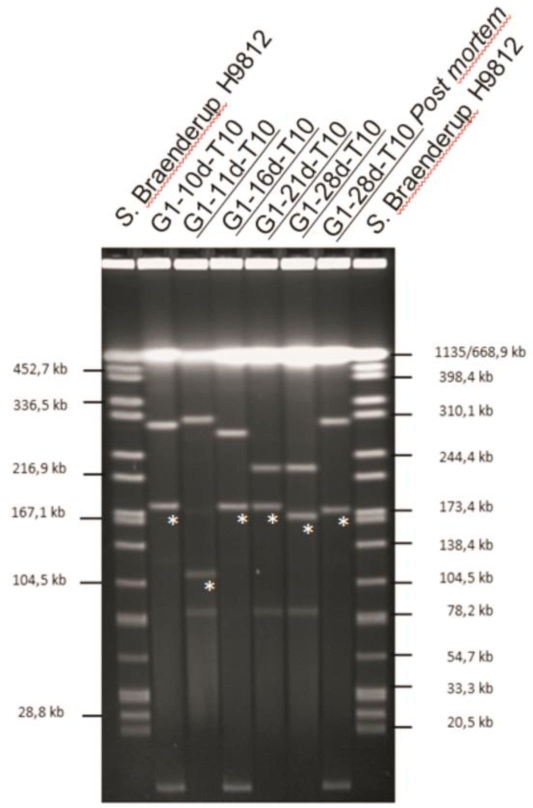

FIGURE S2. S1-PFGE analysis of *S*. Corvallis reisolates from Group 1 in lanes 2-7 with molecular-sized standard, *Salmonella* Braenderup strain H9812 (restricted with XbaI) in lanes 1 and 8. Asterix (*) marked *bla*$_{NDM-1}$ hybridisation. Underlined strains selected for WGS.

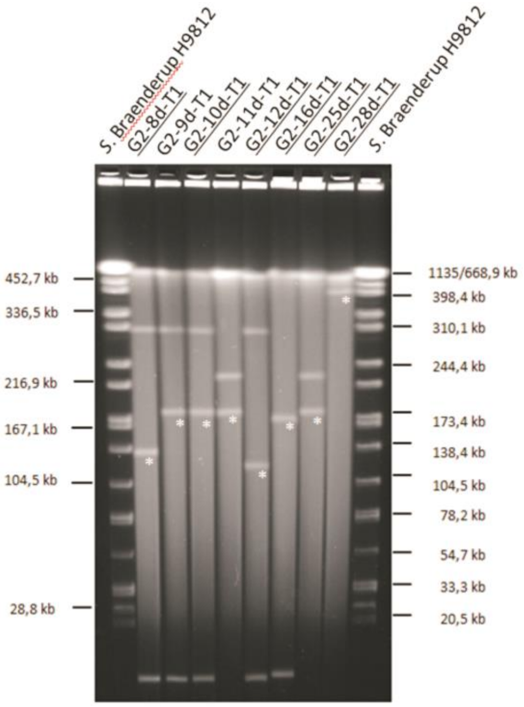

FIGURE S3. S1-PFGE analysis of *S*. Corvallis reisolates from Group 2 in lanes 2-9 with molecular-sized standard, *Salmonella* Braenderup strain H9812 (restricted with XbaI) in lanes 1 and 10. Asterix (*) marked *bla*$_{\text{NDM-1}}$ hybridisation. Underlined strains selected for WGS.

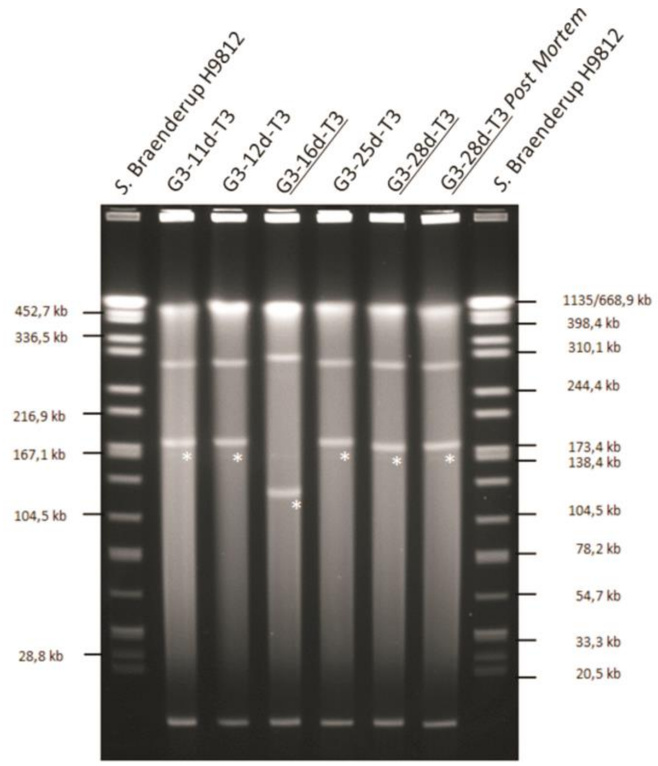

FIGURE S4. S1-PFGE analysis of *S*. Corvallis reisolates from Group 3 in lanes 2-7 with molecular-sized standard, *Salmonella* Braenderup strain H9812 (restricted with XbaI) in lanes 1 and 8. Asterix (*) marked $bla_{NDM\text{-}1}$ hybridisation. Underlined strains selected for WGS.

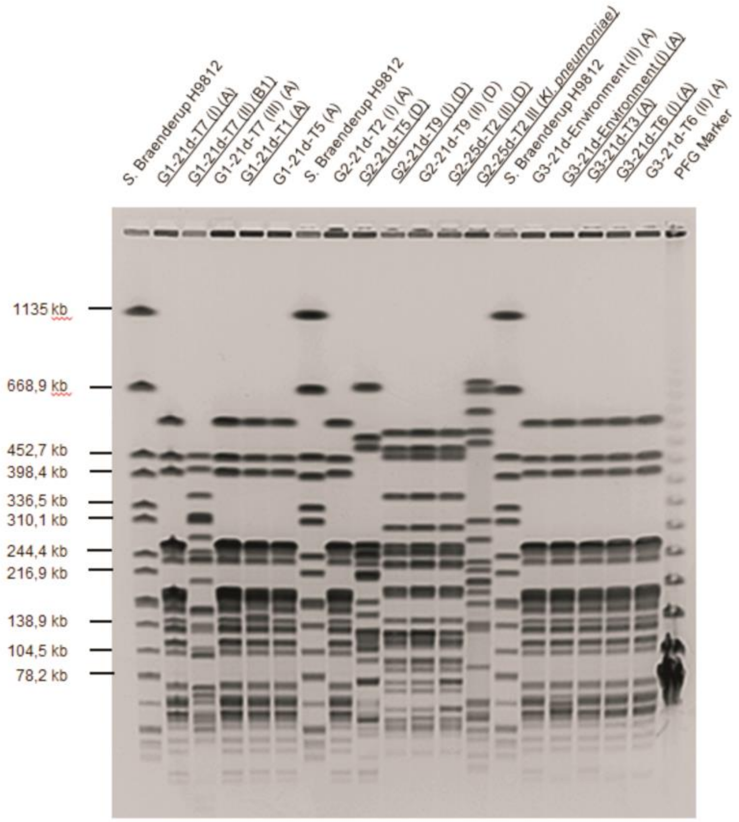

FIGURE S5. XbaI-PFGE analysis of NDM-1 producing Enterobacteriaceae transconjugants from Group 1 (lanes 2-6), Group 2 (lanes 8-13), Group 3 (15-19) with *E. coli* phylogroups (A, B1 and D) designation in brackets and one *K. pneumoniae* strain. Molecular-sized standard *Salmonella* Braenderup strain H9812 (restricted with XbaI) in lanes 1, 7, 14 and MidRange PFG Marker in lane 20. Underlined strains selected for WGS.

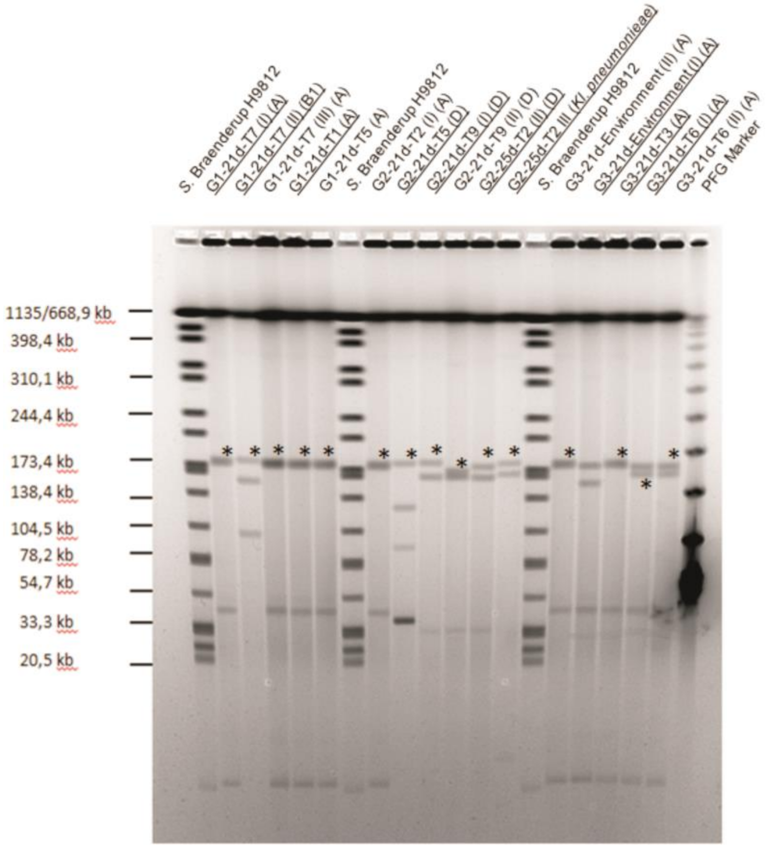

FIGURE S6. S1-PFGE analysis analysis of NDM-1 producing Enterobacteriaceae transconjugants from Group 1 (lanes 2-6), Group 2 (lanes 8-13), Group 3 (15-19) with *E. coli* phylogroups (A, B1 and D) designation in brackets and one *K. pneumoniae* strain. Molecular-sized standard *Salmonella* Braenderup strain H9812 (restricted with XbaI) in lanes 1, 7, 14 and MidRange PFG Marker in lane 20. Asterix (*) marked *bla*$_{NDM-1}$ hybridisation. Underlined strains selected for WGS.

References

1. Fischer J, Schmoger S, Jahn S, Helmuth R, Guerra B. 2013. NDM-1 carbapenemase-producing Salmonella enterica subsp. enterica serovar Corvallis isolated from a wild bird in Germany. J Antimicrob Chemother 68:2954-6.
2. Villa L, Guerra B, Schmoger S, Fischer J, Helmuth R, Zong Z, Garcia-Fernandez A, Carattoli A. 2015. IncA/C Plasmid Carrying blaNDM-1, blaCMY-16, and fosA3 in a Salmonella enterica Serovar Corvallis Strain Isolated from a Migratory Wild Bird in Germany. Antimicrob Agents Chemother 59:6597-600.
3. Nakamura M, Sato S, Ohya T, Suzuki S, Ikeda S. 1985. Possible relationship of a 36-megadalton Salmonella enteritidis plasmid to virulence in mice. Infect Immun 47:831-3.
